# The potential of an optical surface tracking system in non‐coplanar single isocenter treatments of multiple brain metastases

**DOI:** 10.1002/acm2.12866

**Published:** 2020-04-01

**Authors:** Ans C. C. Swinnen, Michel C. Öllers, Chin Loon Ong, Frank Verhaegen

**Affiliations:** ^1^ Maastro Clinic Maastricht the Netherlands; ^2^ Department of Radiation Oncology HagaZiekenhuis Den Haag the Netherlands

**Keywords:** brain metastases, non‐coplanar, open face mask, optical surface tracking, stereotactic radiosurgery

## Abstract

To evaluate the accuracy of a commercial optical surface tracking (OST) system and to demonstrate how it can be implemented to monitor patient positioning during non‐coplanar single isocenter stereotactic treatments of brain metastases. A 3‐camera OST system was used (Catalyst HD™, C‐RAD) on a TruebeamSTx with a 6DoF couch. The setup accuracy and agreement between the OST system, and CBCT and kV‐MV imaging at couch angles 0° and 270°, respectively, were examined. Film measurements at 3 depths in the Rando‐Alderson phantom were performed using a single isocenter non‐coplanar VMAT plan containing 4 brain lesions. Setup of the phantom was performed with CBCT at couch 0° and subsequently monitored by OST at other couch angles. Setup data for 7 volunteers were collected to evaluate the accuracy and reproducibility of the OST system at couch angles 0°, 45°, 90°, 315°, and 270°. These results were also correlated to the couch rotation offsets obtained by a Winston‐Lutz (WL) test. The Rando‐Alderson phantom, as well as volunteers, were fixated using open face masks (Orfit). For repeated tests with the Rando‐Alderson phantom, deviations between rotational and translational isocenter corrections for CBCT and OST systems are always within 0.2° (pitch, roll, yaw), and 0.1mm and 0.5mm (longitudinal, lateral, vertical) for couch positions 0° and 270°, respectively. Dose deviations between the film and TPS doses in the center of the 4 lesions were −1.2%, −0.1%, −0.0%, and −1.9%. Local gamma evaluation criteria of 2%/2 mm and 3%/1 mm yielded pass rates of 99.2%, 99.2%, 98.6%, 89.9% and 98.8%, 97.5%, 81.7%, 78.1% for the 4 lesions. Regarding the volunteers, the mean translational and rotational isocenter shift values were (0.24 ± 0.09) mm and (0.15 ± 0.07) degrees. Largest isocenter shifts were found for couch angles 45˚ and 90˚, confirmed by WL couch rotation offsets. Patient monitoring during non‐coplanar VMAT treatments of brain metastases is feasible with submillimeter accuracy.

## 1 Introduction

While the use of stereotactic radiosurgery (SRS) in patients with a limited number of brain metastases (BM) has been clearly defined, the application of SRS in patients with multiple BM (>4) is still a matter of controversy.^1^ Whole‐brain radiation therapy (WBRT) was traditionally the standard treatment approach, but it is associated with significant side effects, such as cognitive dysfunction (which results in a decreased quality of life), hair loss and fatigue.^1^, ^2^, ^3^ Limiting radiation to the uninvolved brain and obtaining a high probability of local tumor control with a single treatment, are therefore important advantages of SRS over WBRT in patients with 4 or more BM.^4^

Over the years, there has been a lot of technological progress in the way BM is treated with a linear accelerator (linac). Instead of forward planning techniques delivered with static beams or dynamic conformal arcs, an inverse planning technique is used and the beams are delivered with volumetric modulated arc technique (VMAT). Preferably, a single isocenter is used to make the delivery more efficient, thereby reducing the treatment time.^5^ Compared to high precision GammaKnife based treatments of multiple BM, the beam‐on time on a linac is much lower, especially when flattening filter‐free beams are used.^6^, ^7^, ^8^ A crucial aspect is to reduce the GTV‐PTV margin to 1 mm, as the probability of radionecrosis (RN) increases when the V12Gy of the brain exceeds 10 cm^3^.^9^ High‐dose irradiated isodose volume, V22Gy, is also significantly correlated with RN, particularly for patients treated with SRS alone. ^10^ In terms of local control, the randomized study of Kirkpatrick *et al*. showed that there is no difference between the use of 1 mm or 3 mm GTV‐PTV margin.^11^

To treat all BM with such small margin simultaneously, a six degrees‐of‐freedom (6DoF) correction is essential to guarantee a submillimeter setup accuracy. Frameless radiotherapy for treating intracranial lesions has been widely adopted under the guidance of on‐board cone beam CT (CBCT) and a thermoplastic mask system with a 6DoF robotic couch^12^, ^13^, ^14^ or a semi‐robotic couch including manual angle adjustments.^15^

The final step in this progress in the treatment of BM is the introduction of more degrees of freedom by using non‐zero couch angles during treatment planning and delivery. A non‐coplanar technique is not new, but with the introduction of VMAT and image‐guidance techniques (using CBCT at couch 0˚), it became a logical development to maintain to the couch at 0˚, to avoid possible collision problems. The advantages of incorporating non‐zero couch angles in the treatment planning, resulting in better sparing of normal brain tissue, has been published widely.^8^, ^16^, ^17^

In our workflow of non‐coplanar treatments with a standard linac, the patient with BM is immobilized in a thermoplastic mask on the linac equipped with 6DoF couch and with a high‐definition multileaf collimator (MLC). The localization accuracy of the frameless image‐guided system is found to be comparable to robotic or invasive frame‐based radiosurgery systems.^18^ Online CBCT acquisition to verify the patient position is challenging (or even impossible) for a non‐coplanar technique, due to possible collision of the gantry and treatment couch. In order to preserve the GTV‐PTV margin of 1 mm, the patient must be accurately positioned at all times, hence the need for patient monitoring.^19^

The couch rotation offsets from the central axis (CAX) can be quantified using a Winston‐Lutz (WL) test.^20^ This is a commonly used method to localize the isocenter of a linac by correlating the radiation fields directly with the object being irradiated, which is a ball‐bearing (BB) phantom positioned at the center of each radiation field using external lasers and imaged on a piece of film or more recently an electronic portal imaging device.^21^, ^22^ The final position of the BB corresponds to the intersection of the CAX of all sampled radiation fields, in other words, the radiation isocenter.

However, with a good quality control (QC) tool (like the WL test) that guarantees that the couch axis and treatment beam axis are aligned with submillimeter accuracy, it remains questionable whether the accuracy of a non‐coplanar treatment is adequate solely relying on CBCT imaging verification at couch 0˚. A retrospective analysis with 288 SRS brain patients, treated with 1,344 fractions by means of an ExacTrac^®^ system and 6D couch of Brainlab AG (Munich, Germany) has shown that although the patients were fixated with thermoplastic masks, positioning corrections exceeding 1 mm appeared for 42% of beams and exceeding 1° for 9% of the beams.^23^ Further, the longer the treatment delay, the larger the risk of having positioning deviations, it is, therefore, necessary to have a continuous positioning monitoring of the patient in the treatment room while ensuring a short treatment time. Optical Surface Tracking (OST) seems to be a sophisticated and suitable option, as it intends to reduce set‐up errors and provides real‐time non‐invasive monitoring to detect patient movement during treatment, without the use of ionizing radiation.^24^, ^25^, ^26^

The aim in this work is to demonstrate how a commercial OST system can be implemented to monitor patient positioning during treatment for a non‐coplanar single isocenter VMAT technique for multiple BM and to show the feasibility of this system by defining treatment tolerances without compromising the treatment margins.

## 2 Materials and methods

### 2.A Optical surface tracking and patient fixation system

The Catalyst HD™ system is provided by C‐RAD Positioning AB (Uppsala, Sweden). The OST system uses LEDs to project a light of 3 wavelengths (λ = 405nm (blue), λ = 528nm (green), λ = 624nm (red))^1^ onto the patient and a charge‐coupled device camera to detect the light reflected from the patient. Using the information from the reflection the system generates a real‐time 3D surface of the patient, which is compared to a reference surface for verification. The reference surface can either be the body structure (DICOM RT‐STRUCT) from the CT or it can be created directly in the OST system during treatment set‐up. The latter should be applied when using the system on patients (not phantoms), after initial positioning using CBCT at couch 0˚ and used to monitor the patient position through the rest of the treatment. The correspondences between the reference surface to the patient’s real‐time surface are calculated using a non‐rigid algorithm.^27^ The OST system’s calculation of the isocenter shift includes 2 main stages: registration of the reference surface to the live surface and using this registration result to predict the impact on the live surface position by using a volumetric deformable model.^27^ The calculated position inaccuracies are displayed in real‐time in 6 dimensions, including translational and rotational shifts.

**Fig. 1:**
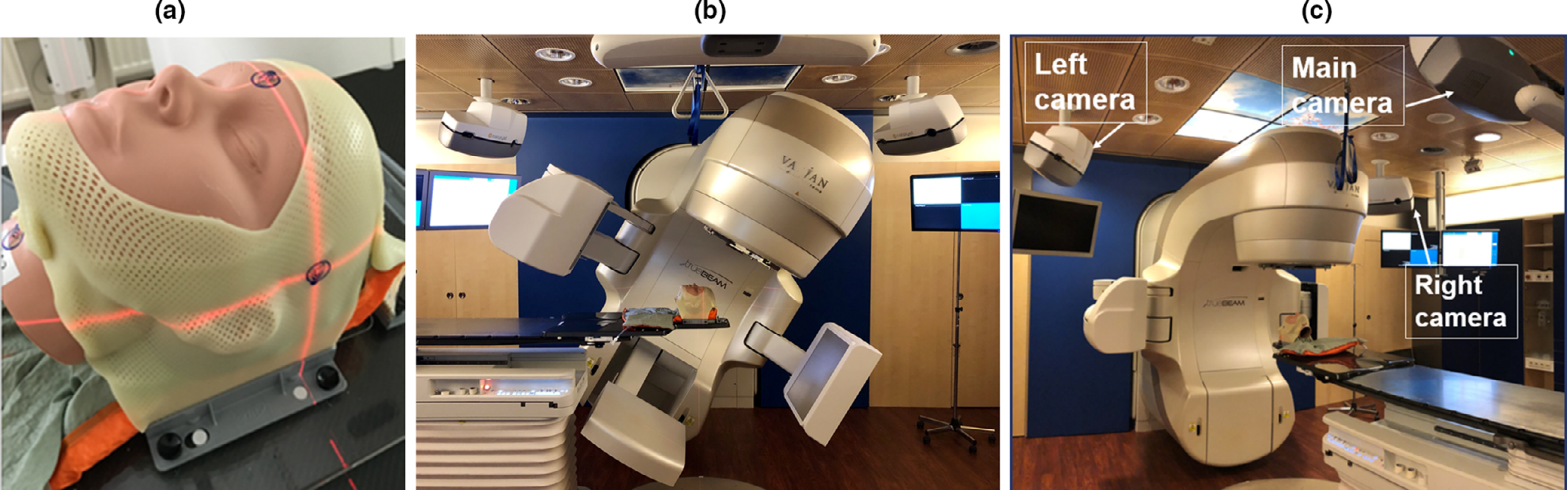
(a) Orfit open face mask and T‐shaped vacuum bag, (b) Catalyst HD^TM^ in kV‐MV setup using the ExaFix‐3 baseplate and (c) in setup at couch 0˚(3 cameras are indicated with arrows)

The advantage of a real‐time monitoring OST system is that it can detect patient movement during treatment, in contrast to the CBCT where the patient position can only be verified during the actual acquisition before the treatment. Furthermore, the OST system, comprising a main camera unit extended with 2 additional camera units with 120˚ angle from the main unit [Fig. 1(c)], has the ability to verify the online patient setup for all couch angles, which makes it highly appropriate for non‐coplanar treatments.

Patient data is imported to the OST system (C‐RAD c4D^TM^ software version 5.4.1) from the treatment planning system (TPS). The exposure time and saturation settings of the 3 cameras can be altered individually to improve the quality of the live patient surface, which makes the system reliable for different skin tones. The scan volume should be adjusted to only include the opening of the mask. A 3 points open face hybrid mask is used in this work, made of 1.6 mm Efficast^®^ and 1.2 mm Nanor^®^ (Orfit Industries, Wijnegem, Belgium). A T‐shape vacuum bag from Orfit is used to support the head, neck, and shoulders [Fig. 1(a)]. The vacuum bag is attached to an ExaFix‐3^®^ baseplate (Macromedics, Waddinxveen, the Netherlands) which locks into the Varian 6DoF couch.

**Fig. 2:**
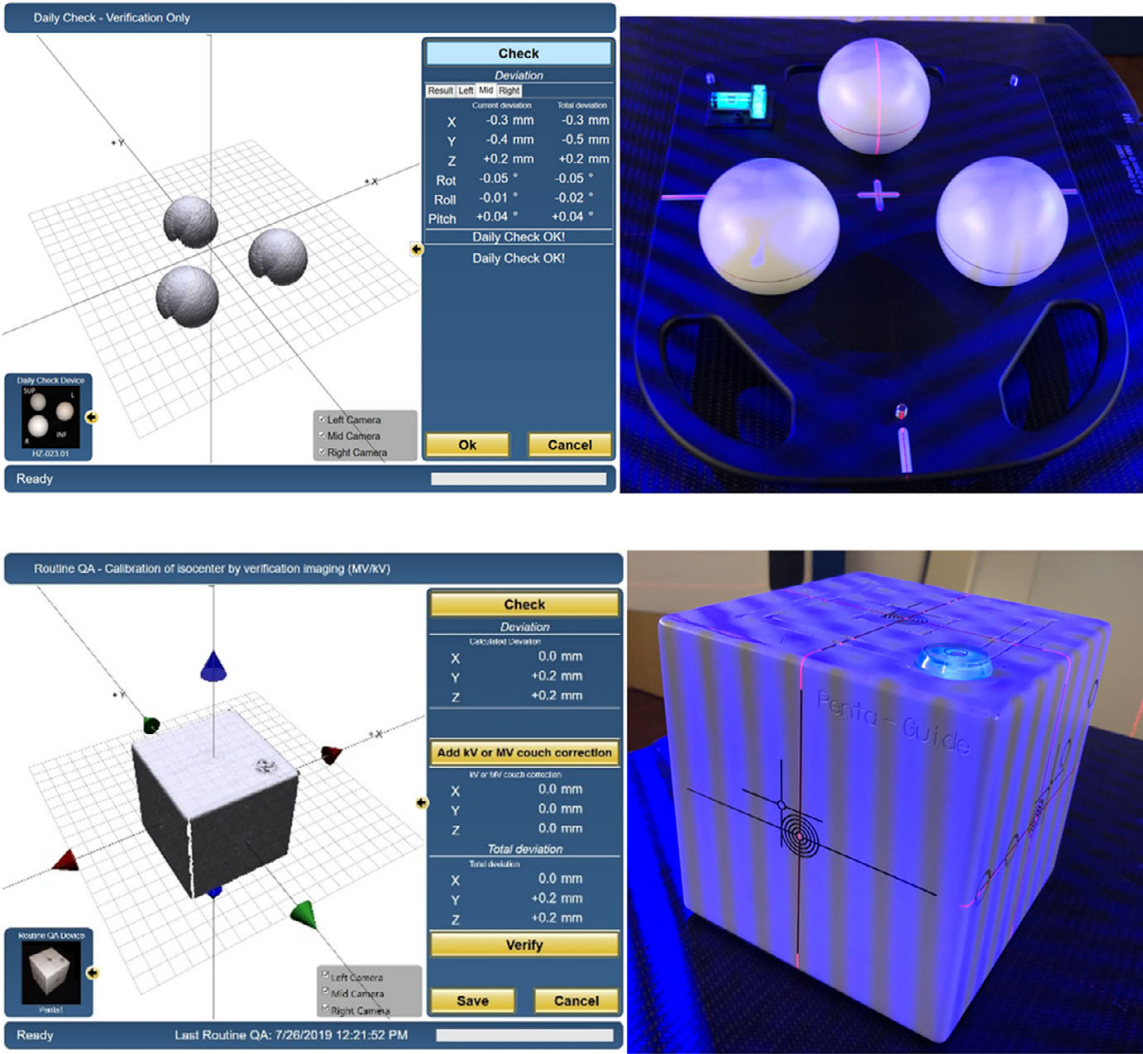
Routine QA procedure consisting of daily QA check for the Catalyst HD^TM^ system (top panel: phantom provided by C‐RAD) and QUASAR^TM^ Penta‐Guide Phantom for the Catalyst HD^TM^ and CBCT system (bottom panel: Modus Medical Devices Inc.)

Prior to any treatment, a Routine QA has to be performed (Fig. 2) and it consists of 2 steps:

1. Daily check phantom provided by C‐RAD, aligned using the room lasers, and directs the focus of the 3 cameras to the isocenter.
2. QUASAR Pentaguide phantom^28^: aligned to the treatment isocenter using CBCT imaging followed by a couch correction based on the match result. This procedure ensures that the Catalyst HD^TM^ is aligned with the CBCT system.

### 2.B Treatment and imaging procedure

Treatment planning was carried out with the Eclipse TPS (Varian Medical Systems, Palo Alto, CA, USA), using the Acuros photon dose calculation algorithm (version 15.5.11). The default beam energy is 6 MV, and calculation grid spacing as well as planning‐CT reconstructed slice thickness for SRS treatments in our clinical practice, are both 1mm. A Varian TrueBeam STx linac equipped with a High Definition 120‐MLC with an inner leaf width of 2.5 mm was used. This type of linac utilizes the jaw tracking technique that keeps the collimator jaws during dose delivery by RapidArc^®^ as close as possible to the MLC aperture, minimizing leakage and transmission through the MLC leaves. As we use a single isocenter technique to treat all BM simultaneously, the field sizes shaped by the jaws are in general always larger than 3 cm^2^ x 3 cm^2^, resulting in acceptable dose calculation accuracy.^29^

A CBCT at couch 0° can detect translational and rotational set‐up errors to be corrected by the 6DoF couch. At couch 270°, it is also possible to verify the patient position using simultaneous kV‐MV imaging and a 2D‐3D match procedure, where the gantry is positioned at 30° and the kV source at 300° [Fig 1(b)]. With a correct definition of the matching box (i.e., around the bone structures of the skull), the 2D‐3D 6DoF matching procedure yields the same result as the 3D‐3D CBCT match.

To assess the coincidence of the imaging centers with the radiation isocenter, the IsoCal is used, which is an automated geometric calibration system for on‐board imaging and MV imaging systems at couch 0°.^30^, ^31^ The comparison of the IsoCal with an independent Winston‐Lutz (WL) method to locate the radiation isocenter has been found to be within 0.4 mm.^30^ In this work, both the IsoCal as a WL test ‐using an in‐house Matlab^TM^ image procession code with 11 combinations of gantry, collimator and couch angles‐ were used as a quality assurance tool. This investigation focused on the WL test results at gantry 0˚ and various couch angle rotations (yaw of 0°, 45°, 90°, 315° and 270°).

### 2.C Stepwise procedure to evaluate the OST system accuracy in combination with open face mask

#### 2.C.1 Setup accuracy evaluation by comparison of OBI with OST

**Fig. 3:**
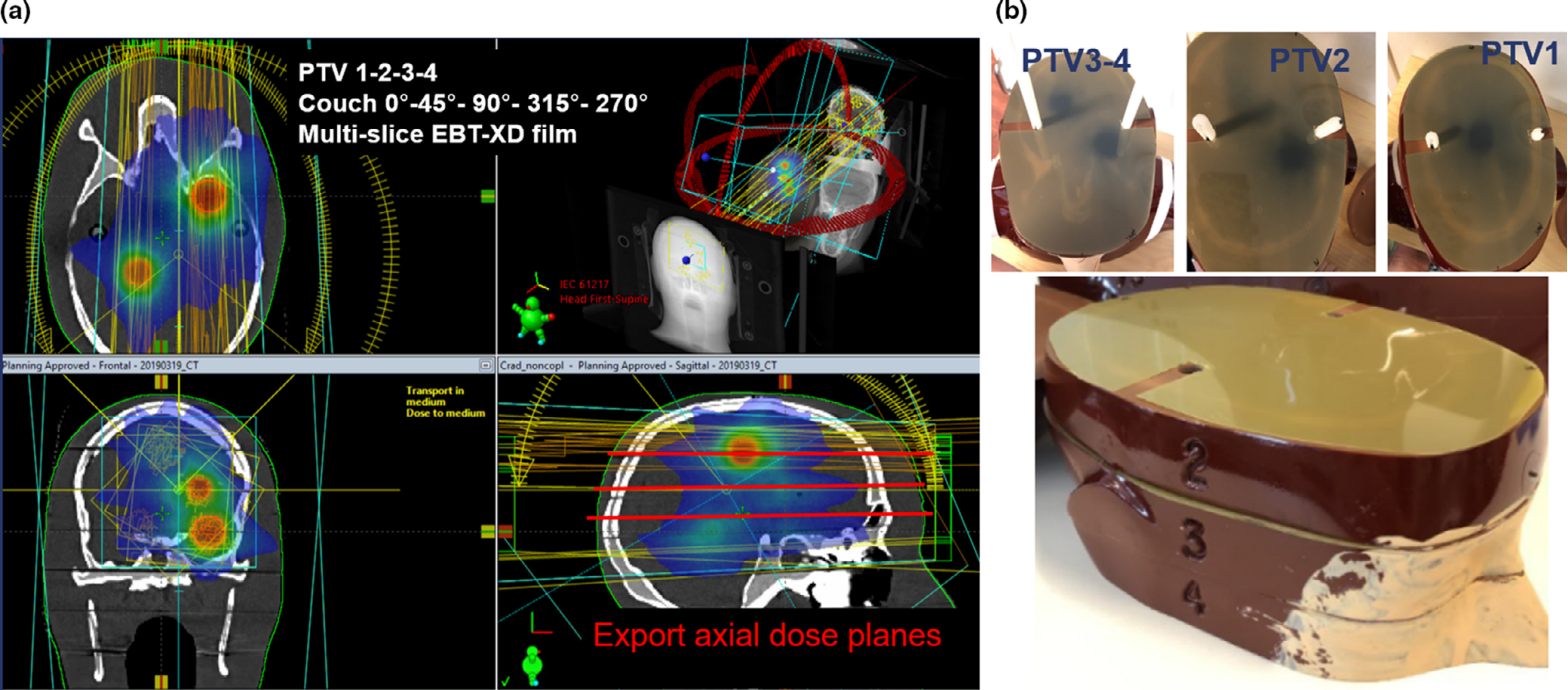
(a) A five arc single isocenter non‐coplanar VMAT plan with 4 PTVs, (b) verified using film dosimetry with EBT‐XD films in the first 3 slices of the Rando‐Alderson phantom head

For an SRS plan with couch 0° and 270°, set‐up accuracy is verified by comparing the agreement between the isocenter shifts calculated by the OST system and the ones suggested after image‐verification with the on‐board kV imaging (OBI). The applied phantom is the Rando‐Alderson (Radiology Support Devices, USA) which is transected into 2.5 cm thick axial slices and incorporates materials to simulate various tissues, bone, and air cavities [Fig. 3(b)]. At couch 0°, a CBCT was made of the phantom head to fine‐tune the setup using a 3D‐3D match procedure, comparing the planning‐CT (pCT) with CBCT (Δ_pCT‐CBCT_). After the correction of rotational and translational errors using the 6DoF couch, a new Catalyst HD^TM^ reference image was made and compared with the external body contour from the planning‐CT scan (Δ_BODY‐OST_). Acquiring the OST reference surface after verification of the position by CBCT eliminates the intrinsic variances between the two systems. To reduce the differences between the original slightly glossy brown phantom color and human skin for the surface scanning, the head phantom was painted in skin color using heavily skin‐tone pigmented make‐up in the area where the face was exposed, resulting in optimized quality of the real‐time surface with more common camera settings. Again, a verification CBCT was made, and the isocenter shift corrections from the 3D‐3D match (Δ_ref‐CBCT_) were compared with the OST measurements (Δ_BODY‐OST_). From this set‐up, the couch was rotated to 270° to check the phantom setup with orthogonal kV‐MV images using a 2D‐3D match procedure, compared with the OST system position shifts.

#### 2.C.2 Dose delivery verification using film

For a case of 4 spherical PTVs simulated in the Rando‐Alderson head phantom, an 8 Gy single isocenter non‐coplanar VMAT plan using couch angles of 0°, 45°, 90°, 315° and 270° was evaluated using GaFchromic^TM^ film measurements (EBT‐XD film batch n^o^ 10231802 (Ashland Inc., Convington, KY, USA)) at measurement depths of 3.5, 6 and 8.5 cm (Fig. 3). The latter depths correspond to the first 3 slices in the head phantom. Although EBT‐XD film is a 2D detector, it provides measurements with a submillimeter spatial resolution to compare with our calculations. The 4 PTVs (with volumes of 2.5, 1.4, 3.8, 1.8 cm^3^) are located in such a way that the center of the PTVs intersect with the measuring planes in order to compare the dose in the middle of the PTV between film and calculation. Before and during delivery of all the beams in the plan, the head phantom was positioned using a CBCT based 6DoF match procedure at couch 0˚ and monitored by OST at all other couch angles. By using a lead fiducial, the position of the films inside the head can be retrieved during analysis. To evaluate the agreement between the absolute measured and calculated dose, FilmQA Pro software (Ashland Inc.) was used for reporting gamma evaluation scores (with agreement criteria of 2%/2 mm as well as 3%/1 mm) and dose deviations in the center of the PTV. In order to put these results into perspective, also a co‐planar plan at couch 0˚ has been delivered to the same phantom set‐up, with a film positioned at 3.5 cm (PTV‐1). The film dosimetry procedure is described in more detail in the appendix.

#### 2.C.3 Mannequin training head

To check whether the Catalyst HD^TM^ is able to accurately visualize the patient at the various couch angles, an experiment was performed with a mannequin training head in the open face mask (i.e., a patient lying motionless) (Fig. 1). The OST system reference surface was captured and the couch was rotated to couch angles 0°, 45°, 90°, 315° and 270°. In theory, if the MV beam and the OST system isocenter are perfectly aligned, any displacement detected by the OST system during couch rotations should only be due to couch rotation drift, assuming the patient has not moved and the OST system is able to accurately visualize the patient at the various couch positions.

#### 2.C.4 Volunteer study using Catalyst HD^TM^

The OST system was tested on 7 Caucasian volunteers (4 males, 3 females, between 22 and 46 years old) fixed with the 3‐points open face mask and vacuum bag in order to determine the accuracy of an SRS treatment monitored by OST in a realistic clinical setting. None of our volunteers had beards or mustaches. However, facial hair can lead to a decrease in light reflection, and consequently loss of information of the face visible to the OST cameras (see the picture of a volunteer’s eyebrows in Fig. 5.). Each volunteer was monitored 3 times as in 3 consecutive treatment fractions for a real patient.

## 3 Results

### 3.A Setup accuracy evaluation by comparison of OBI with OST

For repeated tests with the Rando‐Alderson phantom, the deviations between the isocenter shift suggested after OBI verification and the isocenter shift calculated by the OST system ((Δ_pCT‐OBI_)‐(Δ_BODY‐OST_)) in all translational and rotational directions were always within 0.2° (pitch, roll, yaw) for couch positions 0° as well as 270° and within 0.1 and 0.5 mm (longitudinal, lateral, vertical) for couch positions 0° and 270°, respectively.

### 3.B Dose delivery verification using film

After the CBCT based 6DoF match procedure at couch 0°, the setup of the Rando‐Alderson head is monitored using the OST system at couch angles 315°, 270°, 45°, and 90°.

Deviations between the absolute measured and predicted TPS doses in the center of the 4 PTVs irradiated with a single isocenter non‐coplanar VMAT plan monitored by the OST system are −1.2%, −0.1%, −0.0%, and −1.9% for PTV‐1, PTV‐2, PTV‐3, and PTV‐4, respectively (Table 1). In addition, the deviation between film and TPS doses in the center of PTV‐1 for a co‐planar VMAT plan at couch 0˚ is −1.6%. The daily output fluctuation of the TrueBeam STx linac for 6MV photons was within 0.1%.

**TABLE 1:** The dosimetric agreement between film and TPS doses is presented by the deviations between measured and calculated doses in the center of the 4 PTVs and by a local gamma evaluation criterion of 2%/2mm as well as 3%/1mm for the 4 PTVs irradiated with single isocenter non‐coplanar VMAT and monitored using Catalyst HD^TM^. To put these numbers into perspective, also the results for PTV‐1 irradiated in a co‐planar set‐up (couch 0˚) are given

PTV	D_film_ (Gy)	D_TPS_ (Gy)	Δ (D_film‐_D_TPS_) (%)	Agreement score (2%/2mm)	Agreement score (3%/1mm)
1	9.71	9.82	−1.2%	99.2%	98.8%
1 (co‐planar)	9.15	9.30	−1.6%	99.8%	99.9%
2	9.86	9.87	−0.1%	99.2%	97.5%
3	9.82	9.82	−0.0%	98.6%	81.7%
4	9.11	9.29	−1.9%	89.9%	78.1%

Furthermore, the dosimetric agreement (pass rate) presented by local gamma evaluation criteria of 2%/2 mm and 3%/1 mm both with a cut‐off dose value of 20% (meaning that points with doses below 20% of the maximum are ignored in the gamma analysis) were 99.2%, 99.2%, 98.6%, 89.9% and 98.8%, 97.5%, 81.7%, 78.1% for PTV‐1, PTV‐2, PTV‐3, and PTV‐4, respectively using a rectangular‐shaped region of interest incorporating dose areas from 1.6 to 10 Gy around each of the PTVs (Table 1). Additionally, the dosimetric agreement for the co‐planar plan using the same gamma evaluation criteria (2%/2 mm and 3%/1 mm) was 99.8% and 99.9%, respectively.

### 3.C Mannequin training head

**Fig. 4:**
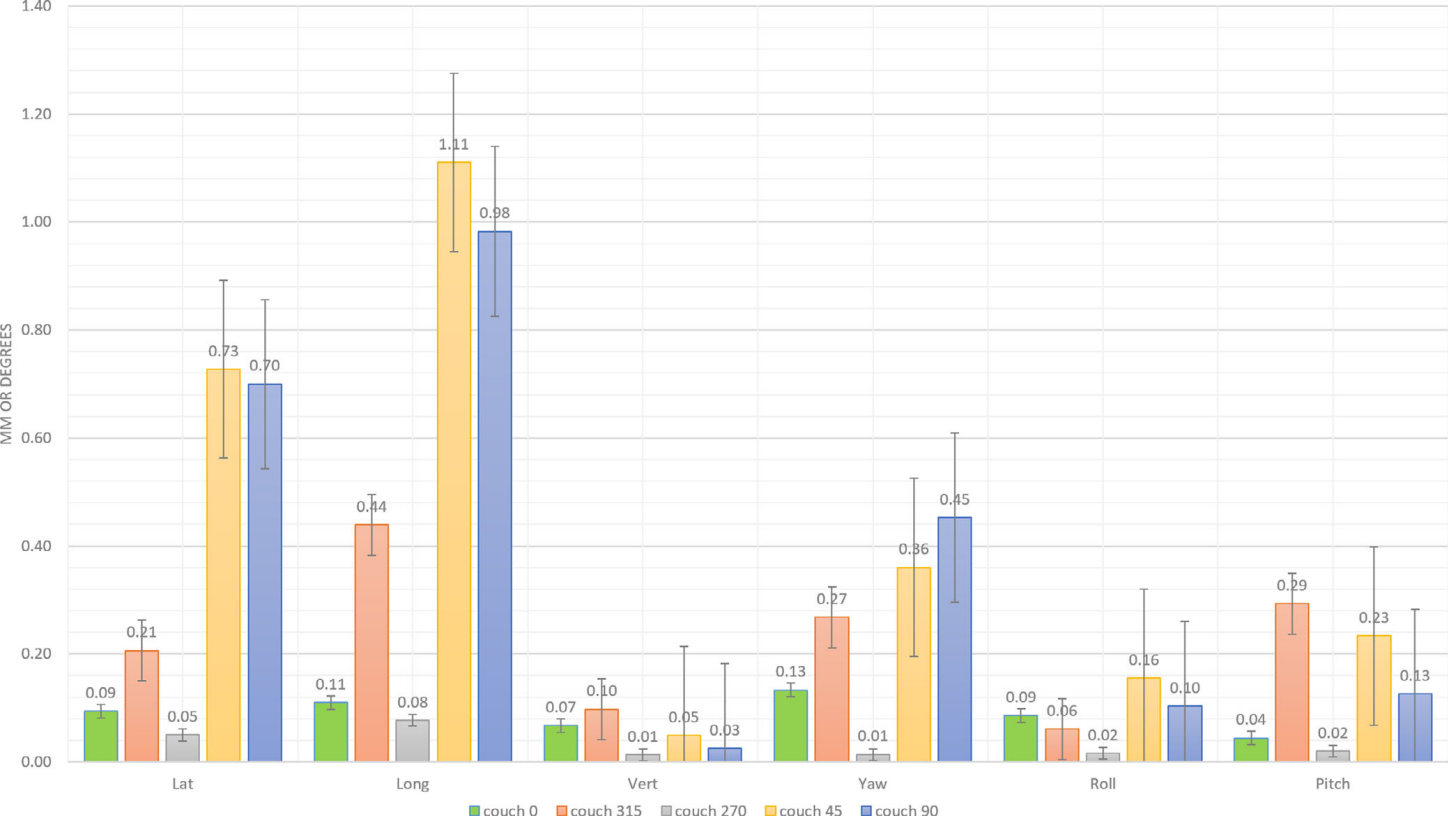
Translational and rotational isocenter shifts (in respectively mm and degrees) of the non‐coplanar treatment (using couch angles 0°, 45°, 90°, 315° and 270°) for the mannequin training head with open face mask, simulating a perfectly immobilized patient

The mean ± SEM values for the translational and rotational shifts for the different couch angles 0°, 45°, 90°, 315°, and 270° obtained from the repeated monitoring sessions are presented in Fig. 4. Deviations larger than 0.5 mm and 0.5˚ are obtained for couch 45˚ and couch 90˚, in a lateral and longitudinal direction.

### 3.D Volunteer study using Catalyst HD^TM^

**Fig. 5:**
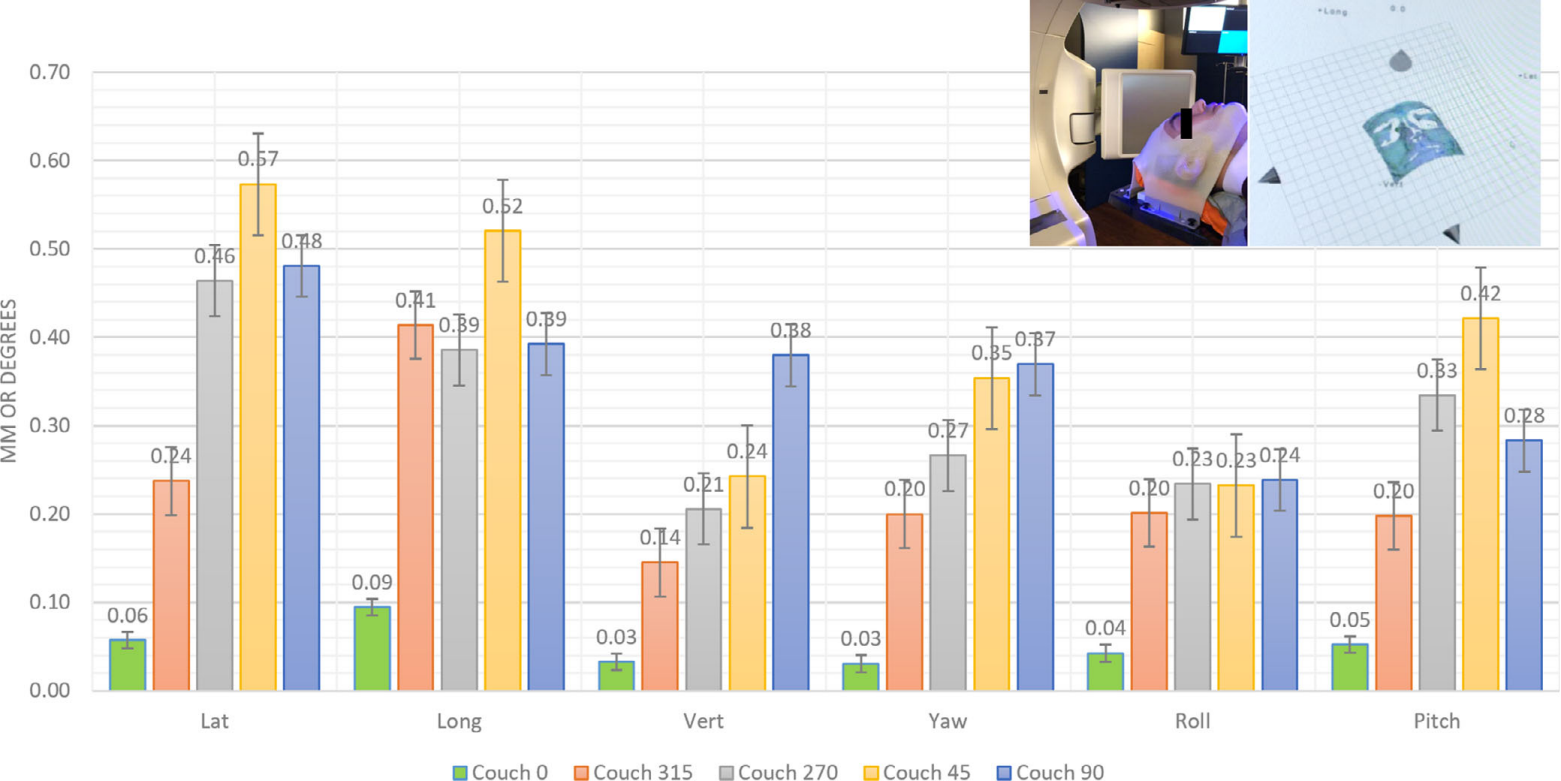
Mean translational and rotational isocenter shifts (in resp. mm and degrees) of the non‐coplanar treatment (using couch angles 0°, 45°, 90°, 315° and 270°) for the population of 7 volunteers with open face mask. A picture of one of the volunteers is depicted in the graph, showing the decrease of information due to facial hair. The error bars represent the standard error of the mean in order to demonstrate how far the sample mean of the data is likely to be from the true population mean

For the 7 volunteers in this study, the mean ± SEM 6DoF isocenter shift values for the couch angles 0°, 45°, 90°, 315°, 270° were within 0.5 mm and 0.5˚ for the different directions (Fig. 5.), except for couch 45˚ in lateral and longitudinal direction.

These results can be correlated with the couch rotation offsets obtained by repeated WL tests performed at the TrueBeam STx over the last 10 months: mean vector deviations between the couch and treatment isocenters at gantry 0° can be found of 0.36 ± 0.15, 0.24 ± 0.09, 0.30 ± 0.14, 0.59 ± 0.11, and 0.60 ± 0.13 (mean ± SD in mm) for couch rotations 0°, 315°, 270°, 90°, and 45°, respectively (Table 2), revealing isocenter deviations larger than 0.5 mm for couch rotations 90° and 45°.

**TABLE 2:** Repeated WL tests over a 10 months period: the deviation for the gantry position at 0˚ and couch positions 0°, 45°, 90°, 315°, and 270° show that the accuracy of the couch exceeds 0.5 mm for couch angles 45˚ and 90˚

WL test	Deviation (mm) at couch 0˚	Deviation (mm) at couch 315˚	Deviation (mm) at couch 270˚	Deviation (mm) at couch 90˚	Deviation (mm) at couch 45˚
1	0.35	0.17	0.25	0.63	0.74
2	0.39	0.17	0.25	0.63	0.55
3	0.39	0.17	0.39	0.63	0.63
4	0.17	0.25	0.17	0.39	0.39
5	0.55	0.17	0.17	0.63	0.63
6	0.25	0.17	0.25	0.63	0.63
7	0.39	0.39	0.55	0.39	0.52
8	0.17	0.35	0.17	0.55	0.39
9	0.35	0.17	0.25	0.63	0.74
10	0.63	0.35	0.52	0.74	0.74
Mean	0.36	0.24	0.30	0.59	0.60
SD	0.15	0.09	0.14	0.11	0.13

## 4 Discussion

With most external beam radiotherapy treatments, an accuracy of ±3 mm is considered desirable and usually achievable. With stereotactic radiotherapy, however, like linac based SRS treatments of patients with multiple BM, somewhat higher accuracy is desired and, with modern techniques, submillimeter accuracy is achievable but requires careful verification.^9^, ^32^ Regarding the technical capability to accurately align the delivery system to the isocenter, current mechanical engineering standards meet this requirement easily.^33^, ^34^ When using frameless, image‐guided SRS (using thermoplastic immobilization masks, CBCT online match procedures, a robotic couch,…), it is necessary to match the imaging isocenter to the mechanical isocenter, which is an achievable goal for standard QA according to AAPM TG‐142 (1 mm/0.5˚).^35^ The IsoCal procedure, as part of the Machine Performance Check designed on TrueBeams to quickly evaluate the machine’s geometric performance^36^ (Varian Medical Systems), guarantees a coincidence between imaging and radiation isocenter within 0.5 mm (namely a built‐in tolerance of 0.2 mm and an action level of 0.5 mm). To verify whether submillimeter accuracy can be achieved between the radiation isocenter and the mechanical isocenter, one would need to perform an end‐to‐end test using image‐guidance and a dosimetric system with the highest spatial resolution, like radiochromic film. Submillimeter accuracy for an end‐to‐end test with a TrueBeam linac has already been demonstrated.^37^ Literature on end‐to‐end testing of a single isocenter VMAT treatment for multiple BM is scarce.^38^, ^39^ When a non‐coplanar technique is applied, the coincidence between radiation and couch rotation isocenter has to be as accurate as possible, as one is generally not able to correct for such errors during treatment delivery. It is thus advisable to have knowledge about the expected range of couch rotation drift using regular QA tests, like a WL test. Further, immobilization devices cannot fully eliminate the intra‐fractional movements of a patient.^23^ In Tryggestad *et al*, a mean intrafractional motion was found to be (1.1 ± 1.2) mm (mean ± SD) using CBCT images, acquired before and after intra‐cranial radiation treatment, to determine movements of the head in a thermoplastic mask.^40^ When the treatment time delay prolongs, the higher the risk of having positioning deviations.^23^ Latter fact also favors the single isocenter technique above a multiple isocenter technique, where an online imaging, as well as the OST procedure, has to be performed for every isocenter separately. This was not part of our investigations but we expect that the use of multiple isocenters has a negative impact on the patient set‐up accuracy.

For a non‐coplanar single isocenter VMAT technique of multiple BM, patient position and movement should be verified continuously at all different couch angles. In this work, we have demonstrated the potential of a commercial OST system as an addition to a conventional linac in monitoring real‐time patient positioning without the use of ionizing radiation and without making concessions to treatment time.

To assess how mechanical, imaging, treatment planning, and radiation isocenter uncertainties combine in a rigid phantom, we performed an end‐to‐end test using multi‐slice film dosimetry in the Rando‐Alderson head phantom. Using a gamma criterion of 2%/2 mm a good agreement between the measurements and predicted TPS doses was found, demonstrating that an accurate dose can be delivered at the correct position. Nevertheless, one should be aware that for very small lesions (<2 cm^3^) this might no longer be the best criterion. As the film has the highest spatial resolution, and since a reduction to 3%/1 mm is possible,^37^ we also looked into this gamma criterion and we still see acceptable agreement scores (Table 1).

From the experiments with the mannequin training head, an increase in OST indicated displacements during couch angle rotations was observed, which ‐in the case of an immobilized patient‐ should only be due to couch rotation drift, as identified by the WL test. However, from Fig. 4. can be seen that random uncertainties in the OST system’s calculation of isocenter shift seem to cumulate on top of the systematic couch rotation drift, such as possible misalignment between radiation beam and OST isocenter (which can be quantified with combined QA measures like IsoCal and Routine QA, which both use CBCT in their procedures). After couch rotation from couch 0˚, it could be that there is less information of the face in the open face mask visible to the OST cameras. To make sure that the observed random errors are very small, we deliberately did a reset of the systematic error due to the couch rotation drift at couch angle 45˚, by performing a refresh of the OST reference surface at this couch angle, which resulted in a perfect match between the live and reference surface (translational shifts <−0.02 mm and rotational shifts <0.1˚). Rotating the couch back to 0˚ gave us the deviation of which we did a reset at couch 45˚. This means that the OST is able to pick up the simulated position deviation.

As not only the position of the couch relative to the couch pedestal has an impact on the couch rotation drift, but also the load on the couch, the results of the volunteers study are important before going clinical. The mean translational isocenter shifts for the 7 volunteers (Fig. 5.) are <0.6 mm, which is in agreement with the average magnitude translation <0.8 mm demonstrated by Lewis *et al.* where an ExacTrac^®^ system (Brainlab, Munich, Germany) is used to monitor the intrafraction patient motion.^41^ They promote to reduce the imaging frequency (to reduce treatment time of the patient in the thermoplastic mask), preserving the necessity of monitoring and correcting intrafractional setup changes to ensure that the dose is distributed according to the treatment plan. A single image acquired pretreatment has found not sufficient to monitor patient motion during SRS.^19^ These findings speak in favor of an OST system as a timesaving non‐invasive solution. In our volunteers study, the average time on the couch took about 12 min, similar to an SRS treatment in our institute. Figure 5. shows that the largest isocenter displacements monitored by the OST system are obtained for couch 45˚ in the lateral and longitudinal direction, which can be correlated to the results of the repeated WL tests (Table 2), with mean translational and rotational isocenter shift values within 0.6 mm and 0.5˚, respectively. From these results, we can suggest that a WL test can deliver valuable information to be taken into account in the treatment planning by avoiding the use of couch angles, where couch rotation drift exceeding a certain tolerance level occurs. Alternatively, when the linac is also equipped with ExacTrac^®^ X‐ray monitoring, the accuracy of the OST system can be easily monitored at any couch position.^41^

Depending on the desired treatment margin, treatment tolerances for the OST system can be defined. In our case, we maintain a more stringent GTV‐PTV margin of 1 mm, so an OST system tolerance of 0.5 mm and 0.5˚ would be feasible for our delivery system, with the exclusion of couch rotations 45˚ and 90˚ which pass the AAPM TG‐142 criteria but demonstrated a less accurate couch rotation coincidence with the radiation isocenter compared to the other couch angles (Table 2).^35^ Therefore, a regular WL test (in our situation with a tolerance of 0.5 mm) should be part of the SRS‐specific QA program of a linac in order to be informed about the capabilities of the SRS delivery system present in the clinic. In order to reduce the time for a (daily) SRS‐specific QA program, future work will be to develop a novel and practical WL test based phantom that integrates several tests, namely measuring the isocenter congruence of imaging systems, radiation beam and couch rotation axis with submillimeter accuracy and also the isocentricity of the OST system.

Other future investigations will include testing similar OST system for brain tumor patients treated with proton therapy on the Mevion S250i (Mevion Medical Systems, Littleton, MA, USA). In our radiotherapy center, a 4 camera system, Catalyst PT^TM^ (C‐RAD, Uppsala, Sweden), has been installed recently in order to monitor the patient on the robotic couch (RoboCouch^®^, Accuray, Sunnyvale, CA, USA) moving in and out the in‐room CBCT (ImagingRing^®^, medPhoton GmbH, Salzburg, Austria) and towards the different treatment positions. As the accuracy in patient set‐up during the various couch movements maybe even more important in brain treatments with proton therapy, we expect the potential of this intrafraction monitoring system to be even higher.

## 5 Conclusion

This work demonstrates a step‐by‐step realization of non‐coplanar single isocenter SRS for multiple BM using the Catalyst HD^TM^. It was shown that submillimeter accuracy by the linac equipped with the OST system can be obtained for these treatments, depending on the type of the delivery system and the tolerances applied during the various SRS‐specific QA procedures. The Catalyst HD^TM^ OST system has the potential to be used as a dedicated patient monitoring tool during complex non‐coplanar high‐precision SRS treatments.

## CONFLICT OF INTEREST

No conflicts of interest.

6

## Note

^1^ While the blue light is the measuring light projected on the patient to determine skin surface coordinates, the green and red light projects mismatches of the reference surface versus the real‐time patient surface directly on the patient skin.

## Appendix 1

In addition to film irradiation, we performed ionization chamber measurements with a cross‐calibrated PTW Farmer chamber (type TN30012, SN 0158) at the depth of 5 cm in RW3 water‐equivalent phantom (SSD 100 cm, 10 cm^2^ × 10 cm^2^, 5 Gy, 6 MV). One film in this set‐up was used for calibration. Also, a blank film from the same batch was applied as a baseline film with 0 Gy.

The films were scanned under a 4 mm glass plate with a flatbed A3‐size Epson 11000XL scanner (Seiko Epson Corp.) with built‐in transparency unit and analyzed using the one‐scan protocol by Lewis *et al.* ^42^ and FilmQA Pro software (version 5.0.5470.35091), a quantitative analysis tool designed for film scanning, pixel value conversion to dose and comprehensive dose analysis (Ashland Inc.).

Additional recommendations mentioned in Mathot *et al.* ^43^ were followed. Due to the anisotropic light scattering in radiochromic films, film orientation must be kept constant, which is in this study “landscape” (where the long axis of the film is perpendicular to the scanner lamp). Further, the reproducibility of film positioning is an important issue due to the non‐uniform scanner response over the scan field perpendicular to the scan direction. The “lateral response effect” causes transmission pixel values to decrease as the lateral distance from the scan‐axis increases. Therefore, we used a ruler template on the scanner glass so that film pieces can be placed in the central scan axis of the scanner bed. Due to the accurate positioning of the film in the center of the scanner and the limited field size, lateral response artifacts, turned out to be negligible.^44^
